# Markerless 3D motion capture for animal locomotion studies

**DOI:** 10.1242/bio.20148086

**Published:** 2014-06-27

**Authors:** William Irvin Sellers, Eishi Hirasaki

**Affiliations:** 1 Faculty of Life Sciences, University of Manchester, Manchester M13 9PT, UK; 2 Primate Research Institute, Kyoto University, Inuyama, Aichi 484-8506, Japan

**Keywords:** Kinematics, Gait, Primate, Bird

## Abstract

Obtaining quantitative data describing the movements of animals is an essential step in understanding their locomotor biology. Outside the laboratory, measuring animal locomotion often relies on video-based approaches and analysis is hampered because of difficulties in calibration and often the limited availability of possible camera positions. It is also usually restricted to two dimensions, which is often an undesirable over-simplification given the essentially three-dimensional nature of many locomotor performances. In this paper we demonstrate a fully three-dimensional approach based on 3D photogrammetric reconstruction using multiple, synchronised video cameras. This approach allows full calibration based on the separation of the individual cameras and will work fully automatically with completely unmarked and undisturbed animals. As such it has the potential to revolutionise work carried out on free-ranging animals in sanctuaries and zoological gardens where ad hoc approaches are essential and access within enclosures often severely restricted. The paper demonstrates the effectiveness of video-based 3D photogrammetry with examples from primates and birds, as well as discussing the current limitations of this technique and illustrating the accuracies that can be obtained. All the software required is open source so this can be a very cost effective approach and provides a methodology of obtaining data in situations where other approaches would be completely ineffective.

## INTRODUCTION

Motion capture, the process of quantifying the movement of a subject, is an essential step in understanding animal locomotion. In many situations it is highly desirable to measure three-dimensional data since the movement of interest cannot be easily reduced to a two-dimensional activity. Even when 2D data are required, for free-ranging animals the requirement for the action to occur perpendicular to the camera axis (Watson et al., 2009) means that many otherwise usable recorded locomotor bouts have to be discarded. In human movement sciences the current state of the art for motion capture is the use of marker clusters on limb segments (Andriacchi et al., 1998), which allow automated, accurate, high speed 3D measurements to be made easily. However, these techniques are much less commonly used in animal studies. Whilst placing markers on a human subject is usually straightforward, in many animal studies this is simply not a practical option, either because the animal does not tolerate the attachment of markers, or because the work is not being performed in a laboratory setting and there is no opportunity to attach markers. Without markers, we need to use a markerless technique, and in the past this has generally meant manual digitisation of video footage. 3D position calculations without markers often have an unacceptably low accuracy because of the need to digitise exactly the same point on multiple cameras, which can be difficult to achieve (Sellers and Crompton, 1994). A further difficulty is that we need to calibrate the 3D space. This is usually achieved by using a calibration object of known dimensions but in many zoo or free ranging settings it may not be easy to do this. In addition the accuracy of 3D calibration is usually dependent on the number of calibration points and their coverage of the field of view (Chen et al., 1994), which further reduces the possible accuracy outside the laboratory. However, recently there has been increasing interest in using non-marker based techniques that rely on photogrammetry, which is seen as having advantages in terms of both potential ease of use and flexibility (Mündermann et al., 2006). Unmarked photogrammetry from multiple, synchronised video cameras has been tried for bird flight studies (Taylor et al., 2008) but in this case it still required considerable manual intervention to assign common points on multiple camera images. However, 3D photogrammetry has now reached the stage where we can extract 3D objects from uncalibrated 2D images. Perhaps the most striking example to date is the “Building Rome in a Day” project, which used images from the Flikr web site (https://www.flickr.com) to generate a 3D model of the whole city (Agarwal et al., 2009).

Automated 3D reconstruction from uncalibrated cameras is essentially a two stage process. Stage one is to reconstruct the camera optical geometry, which requires a number of points that can be identified in multiple images. This reconstruction is achieved using Bundle Adjustment (Triggs et al., 2000). This process assumes an initial set of camera parameters and calculates the reprojection error of the images coordinates onto 3D space. Successive iterations refine the optical parameters to produce a minimal error consensus model where features are located in 3D space and the camera parameters are solved. The ‘bundle’ refers to both the bundles of light rays that leave each 3D feature and converge on the optical centre of each camera, and the fact that the solution is for all the cameras simultaneously. The calibration points can be assigned manually but this is time consuming and potentially not very accurate. However, calibration points can be extracted automatically from many scenes. This is commonly achieved using Scale-Invariant Feature Transform (SIFT) algorithms (Lowe, 1999). These algorithms work by decomposing an image into a set of ‘feature vectors’, which encode areas of the image where there is rapid change of colour and intensity in terms of the underlying morphology. By choosing a suitable encoding system these vectors are largely invariant with respect to the view orientation and can thus be compared between images based on Euclidean distance. Thus in a series of images of the same subject the algorithm can extract large sets of matching features along with a likelihood score for the strength of the match. These can be fed into the bundle adjustment algorithm directly and choosing the correct points can become part of the optimisation task. These techniques rely heavily on rather difficult numerical analysis and only recently have desktop computers become powerful enough for them to become practical options for real-world problems. At the same time considerable work has been done to optimise the required calculations to make this a realistic proposition. Stage two uses the calibrated views to produce a dense point cloud model of the 3D object. There are a number of possible approaches (for a review, see Seitz et al., 2006). Probably the most widespread current approach is patch-based multi-view stereo reconstruction (Furukawa and Ponce, 2010). This approach consists of finding small matching areas of the image, expanding these patches to include neighbouring pixels, and then filtering to eliminate incorrect matches. Remaining patches are then merged to generate a dense 3D point cloud representing the surface of the objects viewed by the cameras excluding areas where the view is occluded or where there is insufficient texture to allow matching to occur.

This photogrammetric approach has gained wide acceptance for producing 3D models of landscapes and static objects in areas such as archaeology (Schaich, 2013) and palaeontology (Falkingham, 2012). However, we wished to ascertain whether it could be used effectively on moving animal subjects to obtain 3D locomotor data by treating individual video frames as still images and using an open source reconstruction work flow. In particular we wanted to know whether the typical resolution of video images and the textural properties of subject animals would allow 3D reconstruction to take place, and if so, to quantify the limitations and inform future work in this area.

## MATERIALS AND METHODS

Photogrammetry works best with high resolution, high contrast, overlapping images of objects with strong textural patterns. To achieve this with video we need to extract sets of simultaneous images from synchronised cameras. The choice of camera is important because we need exact synchronisation to prevent temporal blurring between the individual frames, and we need high quality images with minimal compression artefacts. We used four Canon XF105 high definition video cameras synchronised using an external Blackmagic Design Mini Converter Sync Generator. These cameras have a relatively high data rate (50 Mbps) and a 4:2:2 colour sampling pattern. The cameras were mounted on tripods and directed at the target volume. The separation distance between the cameras was measured using tape measure. A reasonable degree of image overlap was ensured by keeping the angle between the individual cameras to approximately 5 to 10 degrees. To ensure that the motion of the subject was completely frozen, a shutter speed on 1/1000 to 1/500 second was chosen, and to maximise the image quality, the sensor gain was set to 0 dB. This meant that we could only film in relatively bright conditions, and required substantial illumination whilst indoors, which was achieved using photographic floodlights. In addition, exposure, focus and zoom were all locked once the cameras were correctly placed so that the optical parameters remained constant throughout the filming period. Sequences were filmed at either 1080p30 or 720p60 depending on the speed of motion being observed. Interlaced modes were not used to simply data processing and to maximise image quality. The video data from each camera were saved directly to compact flash cards mounted in the cameras in Canon MXF format.

We filmed a number of activities under different conditions. In the laboratory we filmed a Japanese macaque walking on a treadmill. Under free ranging outdoor conditions we filmed Japanese macaques, chimpanzees, and also by chance we managed to film a crow flying through the enclosure. All filming took place at the Primate Research Institute, Kyoto University, and all experimental work was approved through the Animal Welfare and Animal Care Committee following the "Guidelines for Care and Use of Nonhuman Primates of the Primate Research Institute of Kyoto University (3rd edition, 2010)". We have selected a number of use cases that illustrate the capabilities and limitations of the 3D reconstruction technique. To perform the 3D reconstructions we needed to extract the individual frames from the set of cameras as individual, synchronised image files. It proved impossible to start the cameras with frame specific accuracy even though the external genlock means that the frames are themselves always exactly synchronised. This meant that we needed to align the timing of the individual clips after the recording had taken place. This alignment was achieved by first finding a common event that occurred in all the recorded views and noting the frame number associated with that event. In the laboratory experiments this event was artificially generated by dropping at object into the volume of view and seeing when it hit the ground. In the free-ranging experiments we had to rely on identifying a rapid movement made by the animals themselves such as foot or hand strike during locomotion. Once the number of frames of timing offset between the individual cameras was known, we then identified the start and stop frames that marked the intervals within the clips where the animal was doing something we wished to measure. We then extracted the individual frames from each film clip using the open source tool ffmpeg (http://www.ffmpeg.org) and saved them as sequentially numbered JPG files in separate folders, one for each camera.

To perform the 3D reconstruction we initially used *VisualSFM* (http://ccwu.me/vsfm) and would certainly recommend this as an initial step. However, it rapidly became clear that with a large number of frame sets to reconstruct we needed some way of automating the reconstruction. To do this we used python to create a script that would (1) select the synchronous images, (2) apply the feature detector program *vlfeat* (http://www.vlfeat.org) to extract the feature information using the SIFT algorithm, (3) generate lists of possible matches between the images using *KeyMatchFull* from the Bundler package (http://www.cs.cornell.edu/~snavely/bundler), (4) run the program *bundler* (also from the from the Bundler package) to perform the bundle adjustment, and output the camera optical calibration file. Only a single camera calibration file is required for each clip since the cameras do not move. We chose a single image set and checked that the sparse reconstruction produced by *bundler* was correct. We then ran a separate python script that would run the dense point cloud reconstruction program *pmvs2* (http://www.di.ens.fr/pmvs) on all the image sets in the clip using a single camera calibration file for each clip. This script calls *Bundle2PMVS* from the Bundler package to perform *RadialUndistort* on the images and then runs *pmvs2*. The end result is a single folder for each clip containing a numbered list of point cloud files in PLY format, with each point cloud representing the 3D reconstruction from an individual frame set.

Once we had a set of point cloud files, we need a way to measure them. These files are produced at an arbitrary orientation and scale so the first task is to orient the file and apply a suitable scale factor so that any measured data are meaningful. Orientation was done by identifying a vertical direction within the image and rotating the points so that this direction aligned with the +Z axis. Then the horizontal direction of locomotion was defined on this new point cloud, and the point cloud was rotated about the +Z axis until this direction was aligned with the +X axis. The reconstructions use a right handed coordinate system so that +Y will now point to the left hand side of the animal going forward. With the point cloud aligned it was now possible to measure individual points and lines directly from the cloud itself. We could not find any existing tools that could achieve these operations easily and interactively so we wrote a new program called CloudDigitiser (http://www.animalsimulation.org) to allow all these operations to be achieved in a relatively streamlined fashion. This program was written in C++ using the Qt cross-platform toolkit so that it is able to run on Windows, MacOSX and Linux platforms. It allows points, lines and planes to be fitted to groups of points selected using the mouse. It can also calculate and perform the necessary rotations and translations required to define a suitable origin and coordinate system. Once oriented, there are two options for calibration. The easiest option is to measure a known distance within the point cloud and calculate an appropriate scale factor. The cloud should be undistorted so a single scale factor is all that is required. Alternatively, the reconstruction process outputs the positions of the cameras so that their separation can be calculated. Since the actual camera separation has been measure then we can also use this to calculate a suitable scale factor for the cloud. Once a set of calibrated, oriented clouds have been produced, CloudDigitiser allows the user to step between all the cloud files in a particular folder and measure a set of locations off each cloud. These locations can then be exported as a text file for further analysis in any suitable program.

## RESULTS

**Fig. 1.**
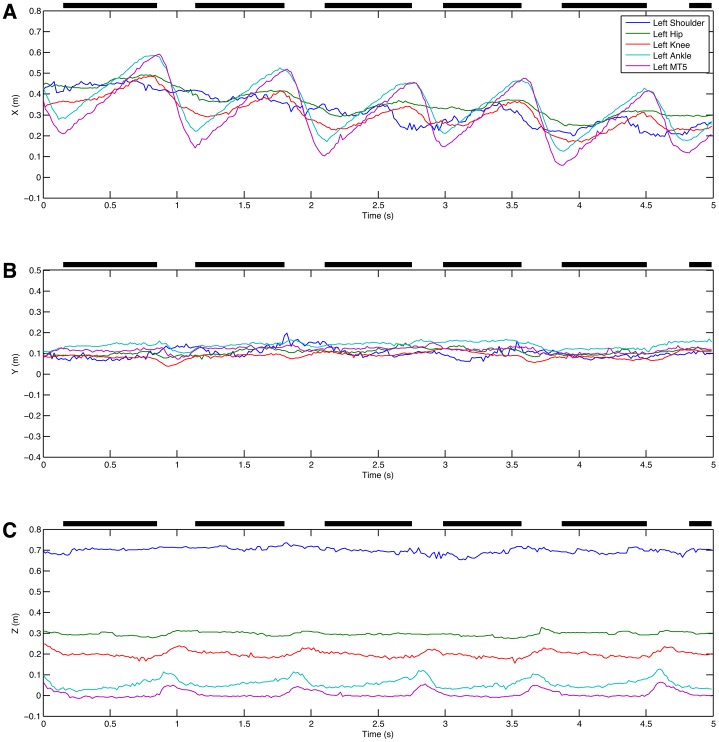
Marker trajectories for a Japanese macaque walking bipedally on a treadmill. (A) X direction (AP). (B) Y direction (lateral). (C) Z direction (vertical).

**Fig. 2.**
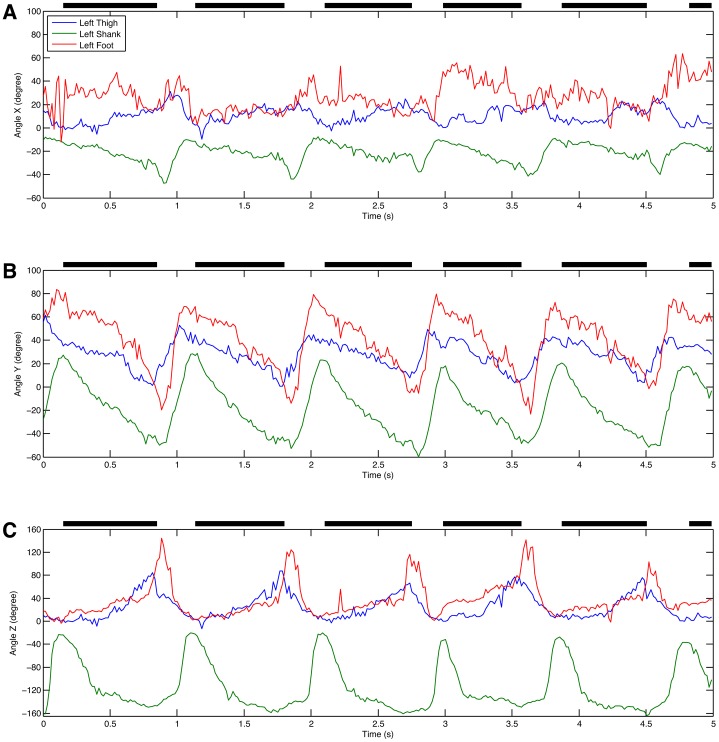
Segment angles for a Japanese macaque walking bipedally on a treadmill. (A) Around X axis. (B) Around Y axis. (C) Around Z axis.

The first example is a laboratory experiment where a male Japanese macaque was trained to walk bipedally on a treadmill. Four cameras were mounted on tripods and positioned at the side of the treadmill, ∼2.5 m from the treadmill and spaced ∼0.35 m apart. The treadmill was brightly illuminated using photographic spotlights enabling a shutter speed of 1/500 s and a gain of 0 dB. The film format was 720p60 giving a frame rate of 60/1.001 frames per second. Orientation and calibration was achieved using the known orientation and dimensions of the wall panels visible in the reconstruction. +X was set as the direction of the treadmill belt, +Z was up and +Y was therefore the right hand side of the monkey. Supplementary material Fig. S1 shows the images from the cameras cropped around the monkey and the 3D reconstruction produced. The field of view of each camera was actually rather larger and included the whole of the treadmill. The 3D reconstructions were analysed by placing virtual markers on the skin over a series of presumed joint centres at the left shoulder, hip, knee, ankle and metatarsal 5 head. CloudDigitiser outputs the marker locations that have been placed as an XML file that can be read into Matlab for further analysis. Fig. 1 shows the 3D positions of the virtual markers over time. Since this is a bipedal walk on the treadmill, it is easy to identify the stance phase by the periods of constant positive X velocity for the metatarsal 5 head virtual marker. The data are quite noisy but this is only to be expected from manually digitised joint centres (e.g. Watson et al., 2009). By comparing the movements of the distal elements in Fig. 1B and Fig. 1C it can be seen that there is actually relatively little lateral movement. However, the picture is clearer if we calculate the angles projected into the X = 0, Y = 0 and Z = 0 planes as shown in Fig. 2. It is now clear that there is appreciable abduction at the hip (Fig. 2A) and that the maximum deviations from vertical coincide with the swing phase indicating that this movement makes an appreciable contribution to ground clearance, although the angular changes occurring in the sagittal plane (Fig. 2B) are much bigger.

**Fig. 3.**
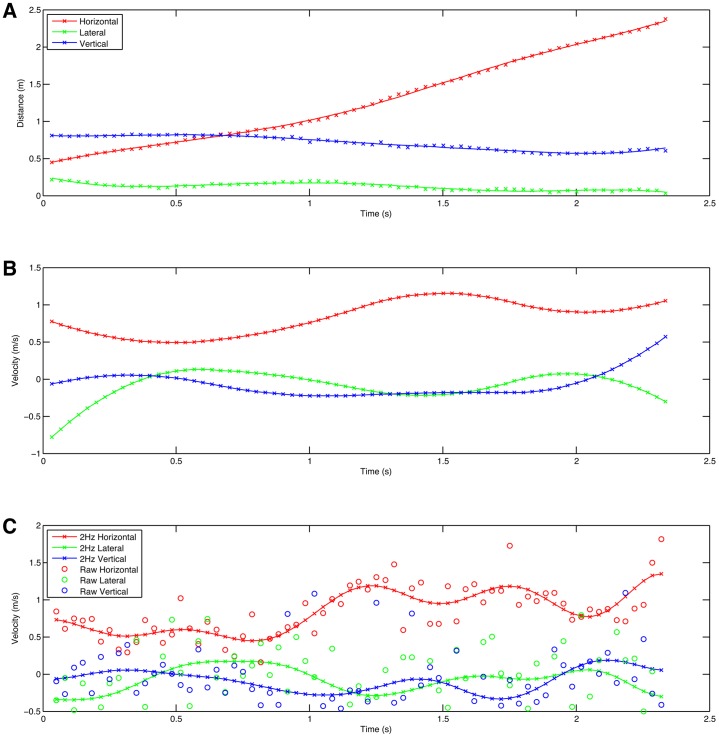
Position and velocity charts for the head marker of a chimpanzee walking bipedally. (A) Position with cubic spline line fit. (B) Velocity derived by differentiating the cubic spline. (C) Velocity derived by linear difference fit to raw (circles) and Butterworth 4 pole low pass bi-directionally filtered data (lines).

The second example shows an adult male chimpanzee walking bipedally on a series of ropes in an outdoor enclosure (supplementary material Fig. S2). This is a fairly typical zoo set up where there is no opportunity to control the location of items within the enclosure, so that there is no control over the movement of the animals. The orientation of the ropes is such that it is impossible to position any cameras perpendicular to the direction of movement, and access to this high location to achieve any in-shot calibration is similarly not possible. In these conditions standard 2D video techniques would only allow qualitative movement descriptions coupled with timing data and this would greatly limit the possible interpretive power. For 3D photogrammetry, we were able to place four cameras on tripods on a convenient balcony some 30 m from the ropes. The camera spacing was set to 2 m between each camera using a tape measure. Filming was done on a bright, sunny day with a shutter speed on 1/1000 s, 0 dB gain, and with the recording format set to 1080p30 and hence a framing rate of 30/1.001 frames per second. Supplementary material Fig. S3 shows the 3D reconstruction produced from the middle of the locomotor bout with the +Z defined from the verticals on the tower, and +X defined from the single rope used as the foot support. The origin location was taken as a point on the support rope close to where it is tied to the tower. Distance calibration was performed using the mean camera separation. The structure of the towers and rope bridges can be clearly seen, as can the body of the chimpanzee. However, there are significant gaps in the reconstruction in areas where there is no textural variation in the fur colour of the animal. To investigate the types of analysis that are possible with these reconstructions we used CloudDigitiser to digitise the estimated locations of the hip, knee, ankle and hand on the right hand side; the ankle and hand on the left hand side; and the head location. Fig. 3 shows the position of the head against time. The positional data are again moderately noisy and although absolute mean velocities can easily be extracted using linear regression (0.85 ms^−1^ in this case), instantaneous velocities are more difficult due to the level of noise. Moderate results can be obtained by spline fitting and differentiation, which is what has been done here (Fig. 3B). Similar results can be obtained using the more typical Butterworth low pass filter (e.g. Pezzack et al., 1977) but because of the noise levels and the relatively low sampling frequency, a very low cutoff frequency (2 Hz) is required (Fig. 3C).

**Fig. 4.**
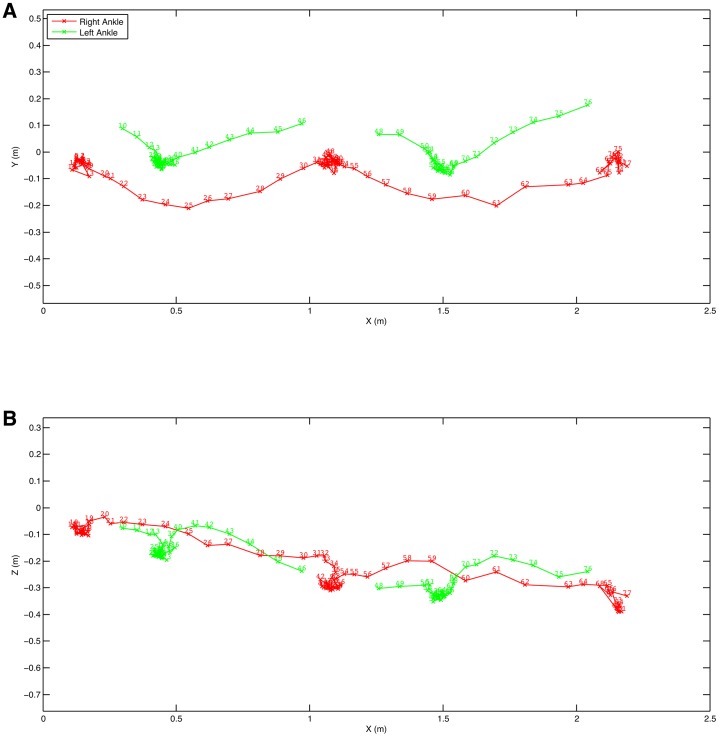
Trajectory of the ankle marker of a chimpanzee walking bipedally. (A) Y (lateral). (B) Z (vertical).

**Fig. 5.**
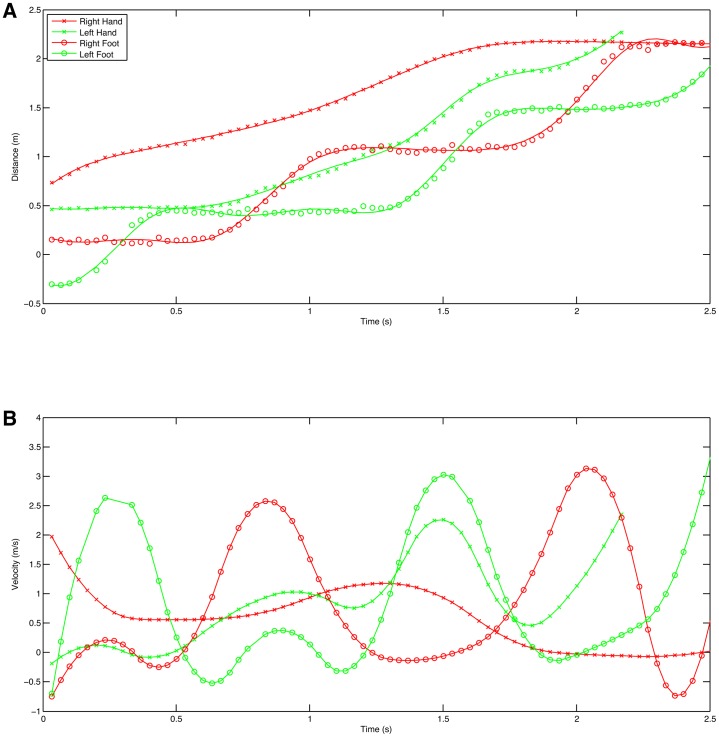
X position and velocity charts for the hand markers of a chimpanzee walking bipedally. (A) X position with cubic spline line fit. (B) Velocity derived by differentiating the cubic spline.

Individual limb movements can also be extracted. Fig. 4 shows the ankle positions as the chimpanzee walks bipedally. These clearly show that the movement during swing phase used to clear the foot from the substrate is a combination of both vertical and lateral deviation with the lateral component being appreciably larger than the vertical component. This lateral component of the movement would be completely missed with a side-on 2D analysis. Fig. 5 shows the hand and foot horizontal positions and velocities. These are interesting because the feet show the clear swing and stance phases as would be expected whereas the hands start with a non-phasic movement as they are slid along the support ropes demonstrating a clear hand-assisted bipedalism (Thorpe et al., 2007), which changes into a more phasic pattern suggesting that the animal switches to something more akin to traditionally described quadrumanuous clambering later in the bout (Napier, 1967).

**Fig. 6.**
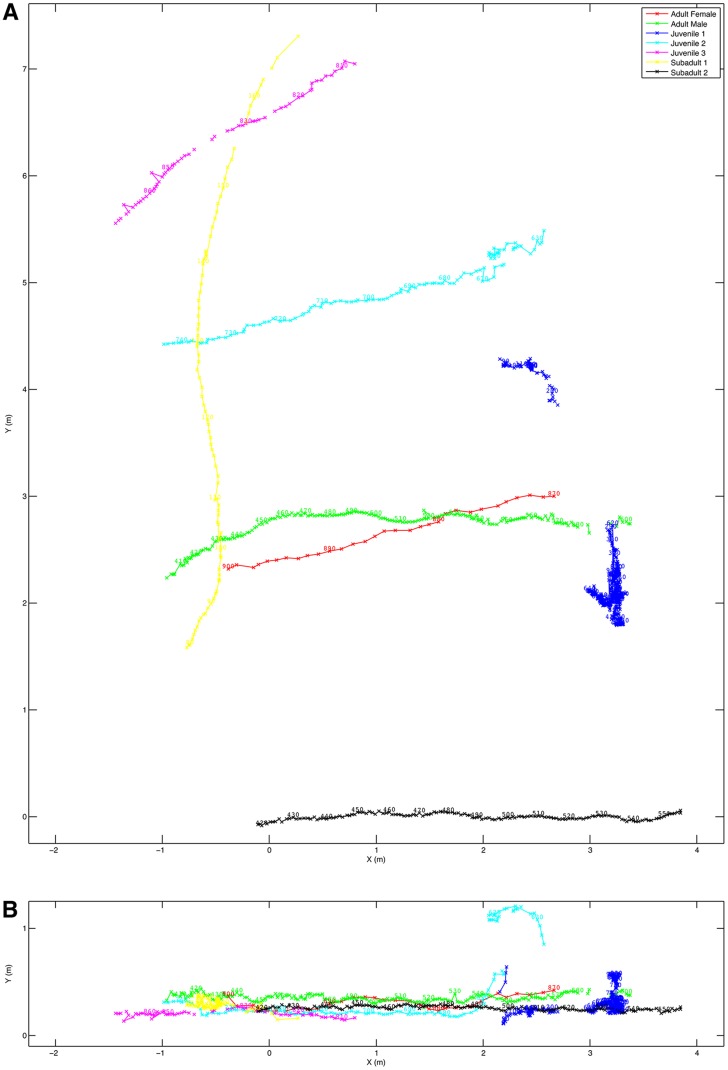
Trajectories of the animals observed in the study area over a 30 s period. (A) Plan view. (B) Side view.

We also wished to test the utility of the 3D photogrammetric approach for multi-animal movement studies. We filmed a group of Japanese macaques on a flat area in their enclosure at a distance of ∼20 m using 4 cameras ∼1.7 m apart. Filming was done on a bright, sunny day with a shutter speed on 1/1000 s, 0 dB gain, and with the recording format set to 1080p30. The camera view is shown in supplementary material Fig. S4 and as can be seen, the camera angle was such that we could only take a steeply raked sideways shot of the area of interest. +Z was defined as the direction perpendicular to the flat surface that the animals were walking across. The choice of X direction in this case was entirely arbitrary and we use the boundary between the gravel and the concrete slope simply because it was a convenient straight line. Distance calibration was performed using the measured camera separation. With animals moving on a flat surface like this, the clearest way of displaying the data is to produce a plan view. However, using 2D approaches, this would require a camera to be mounted above the area of interest, which is rarely possible outside the laboratory. However, as can be seen from supplementary material Fig. S5, the 3D reconstruction can be viewed from any angle desired and whilst the apparent resolution from above is lower, the positions of the animals can be clearly identified. This allows a fully calibrated plan view of the positions of the animals over time (Fig. 6A), and although the area of interest was predominantly flat, it also allows the vertical space usage to be evaluated too (Fig. 6B).

**Fig. 7.**
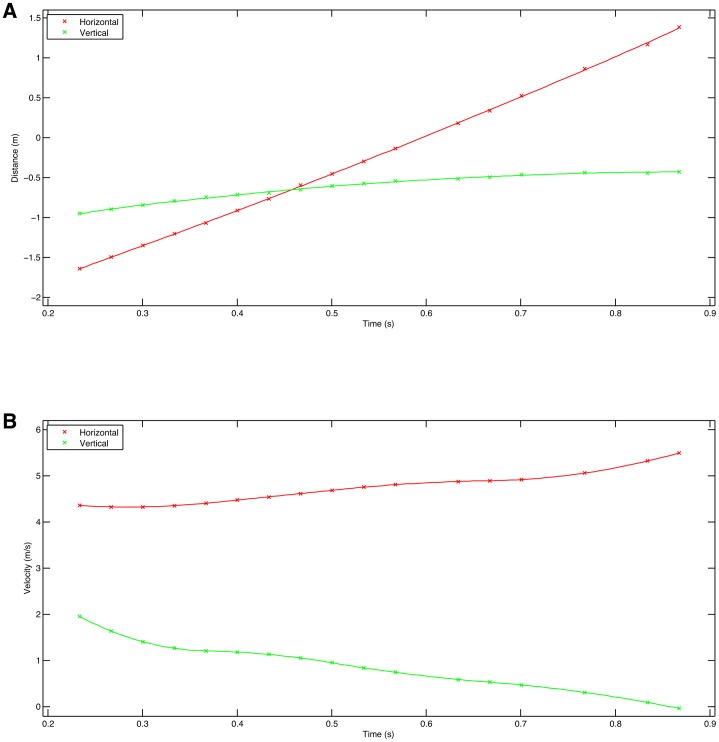
Crow horizontal and vertical head movements. (A) Position with cubic spline line fit. (B) Velocity derived by differentiating the cubic spline.

**Fig. 8.**
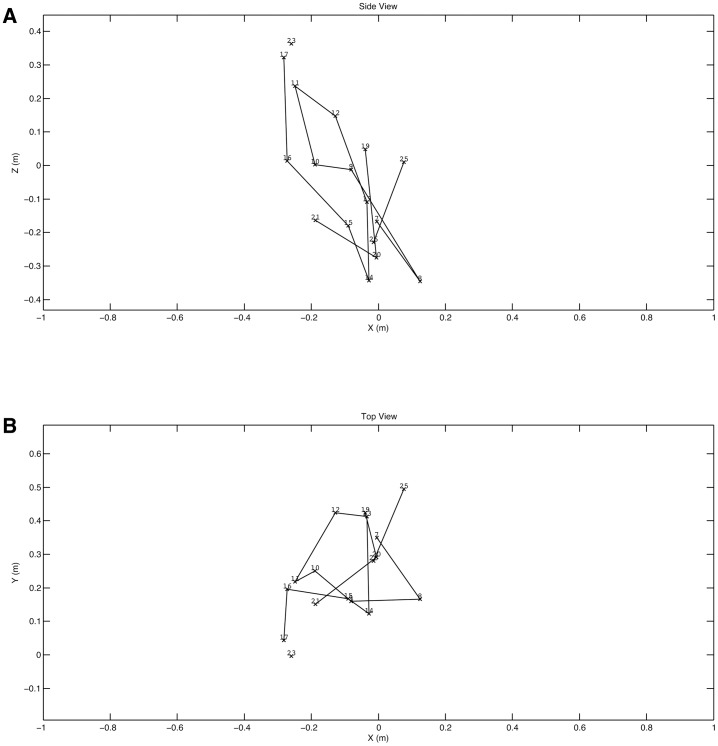
Wingtip trajectory plot with bird moving in positive X direction. (A) Side view. (B) Top view.

Finally, during the course of these experiments to evaluate 3D photogrammetric video on primates, we were able to capture a brief sequence of a crow flying through the field of view and were able to test whether this technique would be useful for studies on flight. The experiment was set up to film Japanese macaques walking along a pole ∼30 m from the observation platform. Four cameras were set up ∼1.7 m apart and we were using a shutter speed on 1/1000 s, 0 dB gain, and with the recording format set to 1080p30. The pole was of known length so this was used directly for calibration, and the vertical poles in the shot were used to orient the +Z axis. Because of the high shutter speed, the bird's motion was frozen very effectively although the relatively low framing rate meant that the temporal resolution of wing movements was comparatively poor (supplementary material Fig. S6). The reconstruction algorithm relies on matching textural patterns in the images. It was therefore pleasantly surprising that an essentially black bird was so well resolved (supplementary material Fig. S7). The rear view (top right, supplementary material Fig. S7) shows the curvature of the wing very clearly. In the point clouds, the +X direction was aligned with one of the horizontal poles but for the analysis the bird's direction of motion was used to define the +X direction. This was done by fitting a line to the sequential positions of the bird's head and rotating the measurements around the Z axis until this fitted line was parallel to the X axis. This allowed all the extracted measurements to be relative to the horizontal direction of travel. Fig. 7A shows the horizontal and vertical flight paths and by fitting a straight line we can calculate that the mean speed over the ground is 4.74 ms^−1^ and the mean rate of ascent is 0.82 ms^−1^. The instantaneous velocity can also be calculated by differentiation (Fig. 7B) although again care must be taken with data smoothing. Obtaining values such as these from free flying birds is extraordinarily difficult. Similarly the wing 3D trajectory can be obtained by placing virtual markers on the wing tip. Wingtip trajectories are commonly recorded in wind tunnel experiments (e.g. Tobalske and Dial, 1996) but obtaining equivalent information on free flying birds is much more challenging and allows us to obtain information from non-steady state activities such as turning. The lateral and side views of the wingtip trajectories are shown in Fig. 8.

## DISCUSSION

The four examples presented demonstrate the utility of 3D video photogrammetry. The technique obviously works best in a laboratory situation where lighting can be used to maximise the contrast on the surface of the animal. Like any video-based technique, it benefits from situations where the movement of the subject can be restricted so that as much as possible of the field of view can contain useful information. This maximises the resolution and produces the highest quality data. Even so, it is clear that there is considerable resolution loss moving from the original 2D images to the 3D reconstruction (supplementary material Fig. S1), and the reconstruction has gaps that mean that marker positions have to be interpolated. On the plus side, the 3D reconstruction removes any parallax errors from the data and these can cause significant errors in 2D data collection when it is not possible to move the cameras to a large enough distance to allow the effects of distance changes to be ignored. If the subject is amenable to the attachment of motion capture markers the accuracy and ease of use would be improved, but if markers are an option then a standard commercial 3D motion capture system will produce better data far more efficiently than the photogrammetric approach presented here. However, there are many laboratory situations such as bird (Tobalske and Dial, 1996) or insect (Ellington, 1984) flight where attaching markers is difficult or may affect the outcome of the experiment, and this is where video photogrammetry provides a viable option for obtaining 3D data.

The technique really comes into its own outside the laboratory environment. The data presented here on chimpanzee bipedalism would not have been possible to obtain using traditional techniques. It is often the case that calibration is impossible and obtaining any quantitative kinematic data requires time consuming and relatively inaccurate approaches such as surveying the enclosure (Channon et al., 2012) or using parallel lasers (Rothman et al., 2008). 3D video photogrammetry is self-calibrating based on the separation of the cameras so it will always generate absolute magnitudes. The fact that the data produced are 3D means that a much greater proportion of performances can be measured successfully, which is essential for relatively rare occurrences such as bipedalism. It also means that the analysis can take place in 3D. It is certainly true that many locomotor studies are restricted to 2D, not because the phenomenon being studied is well approximated by a 2D model, but because obtaining 3D data is much more difficult. Thus the observation made here that the foot movement laterally in swing phase is greater than the movement vertically would not have been apparent with a 2D technique. In addition, because of practical requirements in terms of laboratory facilities or camera placement, many chimpanzee locomotor studies (e.g. D'Août et al., 2004; Sockol et al., 2007; Watson et al., 2011) are terrestrial and this means that important features of their locomotor system are not being adequately assessed. The flexibility of 3D photogrammetry means that there are many more opportunities for recording the actual kinematics of animals performing complex locomotor activities in naturalistic enclosures.

This is equally the case when considering group interactions. Outside the laboratory there is little choice in where cameras are placed and without a 3D reconstruction it is not possible to get good quality spatial data from lateral camera views. With a self-calibrating 3D system it is possible to compensate for sub-optimal camera positions and to generate an accurate spatial view from any desired direction (Fig. 6). This opens the possibility of doing a full, quantitative spatial analysis of any group-living animal, which would then allow the quantitative testing of model predictions (Hamilton, 1971; De Vos and O'Riain, 2010) and provide inputs for a range of spatial studies such as agent-based modelling (Sellers et al., 2007) and enclosure use (Ross et al., 2011). Furthermore, because this technique demonstrably works on birds in flight (supplementary material Fig. S7), groups do not have to be restricted to a plane, and more complex 3D flocking behaviours can potentially be analysed (Davis, 1980), which may provide a more flexible approach than the current stereoscopic techniques (Ballerini et al., 2008).

However, 3D video photogrammetry is not without its own difficulties. The process of 3D reconstruction reduces the apparent resolution of the video images considerably and this means that detail is much harder to see and it becomes more important that the movement of interest fills the reconstruction volume (supplementary material Fig. S1). We would suggest that the advent of affordable 4K cameras may well prove very useful in this context to maintain a desirable reconstruction accuracy. Photogrammetric 3D reconstruction also requires high quality images to work from. We found that in conditions of poor light or low contrast, the algorithms were much less successful and reconstructions often failed completely. In addition it was important that there was enough texture in the shared fields of view for the bundle adjustment to calibrate the cameras. This was generally the case, but could fail if, for example, there was very little background information because the animal was positioned against the sky, or against a featureless (or indeed a very regular patterned) wall. In laboratory conditions, getting the lighting correct was important, and a bright side light to enhance the shadows created by the fur proved to be useful. Similarly it was helpful if there was plenty of static texture in the field of view – quite the reverse of the plain backgrounds normally used in video-based motion capture approaches. The reconstruction quality is quite variable and there is a tradeoff between the completeness of the reconstruction and noise level. Getting the exposure level correct so there are no areas where the subject is over-saturated or completely dark is also an important factor. Using high dynamic range cameras would help this, but it is certainly a problem when particular areas of the animal's body are not reconstructed due to a lack of texture. In general the requirements for high quality images mean that this technique would benefit from greater skill as a videographer and higher quality cameras than would normally be considered necessary.

The reconstruction process itself is computationally demanding. On a single processor desktop it can take about 30 minutes to reconstruct a single frame set. With multiple processors it is relatively easy to process multiple framesets simultaneous, and some aspects of the reconstruction are implemented to take advantage of multiple processor environments and graphics card processing. However, it can still take a very long time to process a set of clips. The real disadvantage of this is that it may not be possible to check the quality of the reconstruction whilst still on site, and any alterations to data collection protocols may have to wait until the 3D reconstructions have been evaluated. The 3D reconstruction can work with any type of camera but high speed filming would necessarily lead to more images to process and even greater time and computational demands. Currently the computational tools available are not especially easy to use. There are some commercial tools available but these generally do not provide the batch capabilities required to process sets of video images. However, there is a great deal of interest in this research area both from academics and commercial interests so we would predict that there will be appreciable software advances in the next few years. In particular *VisualSFM* now includes batch processing capabilities and uses GPU-based acceleration so might be preferable for new users although the time consuming part of the process is still the *pmvs2* step. Another issue is file size. A great deal of work has been applied to video files so that they can be efficiently compressed and thus reduced to manageable sizes with minimal quality loss. As far as we are aware, no such lossy compressed file formats exist for 3D models. Each individual PLY file can be as large as 30–40 MB depending on the field of view and any objects in the background. Thus a 10 second clip at 60 fps can take up almost 25 GB of space. Thus having adequate storage space is an important consideration.

In terms of analysis, our *CloudDigitiser* tool makes manual measurement of specific points on the body reasonably straightforward. Since we are not restricted to pre-assigned marker locations, there is a great deal of flexibility to choose how the data are analysed after the experiment. However, we feel that the sort of data obtained by this technique would probably benefit from non-traditional forms of analysis. Obviously if the locations of particular points are the direct research goal then the approach presented here is ideal, and certainly straightforward. Often, though, these points are used as methods for generating other derived properties such as joint excursion angles and positions of centres of mass. We would suggest that when working from point cloud surface data, there are better approaches. For example, the angle of a limb segment may be better measured by fitting a line to the 3D body surface, and joint angles calculated directly from this. Similarly, with a point cloud, the centre of mass can best be estimated by fitting a segment outline to the available data. Probably the best option would be to fit the 3D outline of an articulated model of the subject animal to the complete surface data. These approaches would need to be customised for each particular species, which is a great deal of work, but they should provide very much higher quality kinematic data and cope well with the issues associated with blank patches where the reconstruction has failed due to lack of visible texture. In addition, 3D video photogrammetry can provide data that are not normally available. By recording complete surfaces and volumes it becomes possible to consider soft-tissue movements in much greater detail and for particular studies, such as locomotion in obese animals, this could be invaluable. Another advantage of photogrammetric approaches is that they work at any scale, and in any medium. It would therefore be possible to adapt these techniques to perform 3D measurements on very small animals such as insects or to reconstruct fish movements underwater. In addition the point clouds produced may allow novel analysis of a wide range of invertebrates without rigid skeletons. One possible advance is that it is not necessary to keep the cameras fixed. The reconstruction does not need to use any information from previous frames so cameras could be panned and zoomed as necessary to keep the target in the field of view. This would potentially allow a much greater resolution and allow animals to be followed over much greater distances. However, there would then be a need to reassemble multiple 3D reconstructions, which would be computationally challenging. We would also predict that there are likely to be considerable software advances in this area, and with improved quality and reliability, multi-camera 3D reconstruction will become an important archive technique to preserve the forms and locomotion for the sadly increasingly large number of endangered species.

### Conclusion

Markerless 3D motion capture is possible using multiple, synchronised high definition video cameras. It provides a way of measuring animal kinematics in situations where no other techniques are possible. However, there are still a number of technical challenges that mean that marker-based systems would still currently be preferred if they are feasible. However, we would predict that this approach is likely to become more prevalent as both hardware and software improve.

## Supplementary Material

Supplementary Material

## References

[b1] Agarwal S. Snavely N. Simon I. Seitz S. M. Szeliski R. (2009). Building Rome in a day. Proceedings of the International Conference on Human Vision Kyoto.

[b2] Andriacchi T. P. Alexander E. J. Toney M. K. Dyrby C. Sum J. (1998). A point cluster method for in vivo motion analysis: applied to a study of knee kinematics. J. Biomech. Eng. 120, 743–749 10.1115/1.2834888 10412458

[b3] Ballerini M. Cabibbo N. Candelier R. Cavagna A. Cisbani E. Giardina I. Lecomte V. Orlandi A. Parisi G. Procaccini A. (2008). Interaction ruling animal collective behavior depends on topological rather than metric distance: evidence from a field study. Proc. Natl. Acad. Sci. USA 105, 1232–1237 10.1073/pnas.0711437105 18227508 PMC2234121

[b4] Channon A. J. Usherwood J. R. Crompton R. H. Günther M. M. Vereecke E. E. (2012). The extraordinary athletic performance of leaping gibbons. Biol. Lett. 8, 46–49 10.1098/rsbl.2011.0574 21831879 PMC3259959

[b5] Chen L. Armstrong C. W. Raftopoulos D. D. (1994). An investigation on the accuracy of three-dimensional space reconstruction using the direct linear transformation technique. J. Biomech. 27, 493–500 10.1016/0021-9290(94)90024-8 8188729

[b6] D'Août K. Vereecke E. Schoonaert K. De Clercq D. Van Elsacker L. Aerts P. (2004). Locomotion in bonobos (Pan paniscus): differences and similarities between bipedal and quadrupedal terrestrial walking, and a comparison with other locomotor modes. J. Anat. 204, 353–361 10.1111/j.0021-8782.2004.00292.x 15198700 PMC1571309

[b7] Davis J. M. (1980). The coordinated aerobatics of dunlin flocks. Anim. Behav. 28, 668–673 10.1016/S0003-3472(80)80127-8

[b8] De Vos A. O'Riain M. J. (2010). Sharks shape the geometry of a selfish seal herd: experimental evidence from seal decoys. Biol. Lett. 6, 48–50 10.1098/rsbl.2009.0628 19793737 PMC2817263

[b9] Ellington C. P. (1984). The aerodynamics of hovering insect flight. III. Kinematics. Philos. Trans. R. Soc. B 305, 41–78 10.1098/rstb.1984.0051

[b10] Falkingham P. L. (2012). Acquisition of high resolution three-dimensional models using free, open-source, photogrammetric software. Palaeontologia Electronica 15, 1T.

[b11] Furukawa Y. Ponce J. (2010). Accurate, dense, and robust multiview stereopsis. IEEE Trans. Pattern Anal. Mach. Intell. 32, 1362–1376 10.1109/TPAMI.2009.161 20558871

[b12] Hamilton W. D. (1971). Geometry for the selfish herd. J. Theor. Biol. 31, 295–311 10.1016/0022-5193(71)90189-5 5104951

[b13] Lowe D. G. (1999). Object recognition from local scale-invariant features. Proceedings of the International Conference on Computer Vision, Corfu, Greece 1150–1157 IEEE Computer Society.

[b14] Mündermann L. Corazza S. Andriacchi T. P. (2006). The evolution of methods for the capture of human movement leading to markerless motion capture for biomechanical applications. J. Neuroeng. Rehabil. 3, 6 10.1186/1743-0003-3-6 16539701 PMC1513229

[b15] Napier J. R. (1967). Evolutionary aspects of primate locomotion. Am. J. Phys. Anthropol. 27, 333–341 10.1002/ajpa.1330270306 4968059

[b16] Pezzack J. C. Norman R. W. Winter D. A. (1977). An assessment of derivative determining techniques used for motion analysis. J. Biomech. 10, 377–382 10.1016/0021-9290(77)90010-0 893476

[b17] Ross S. R. Calcutt S. Schapiro S. J. Hau J. (2011). Space use selectivity by chimpanzees and gorillas in an indoor-outdoor enclosure. Am. J. Primatol. 73, 197–208 10.1002/ajp.20891 20938928 PMC7141771

[b18] Rothman J. M. Chapman C. A. Twinomugisha D. Wasserman M. D. Lambert J. E. Goldberg T. L. (2008). Measuring physical traits of primates remotely: the use of parallel lasers. Am. J. Primatol. 70, 1191–1195 10.1002/ajp.20611 18767123

[b19] Schaich M. (2013). Combined 3D scanning and photogrammetry surveys with 3D database support for archaeology and cultural heritage. A practice report on ArcTron's information system aSPECT3D. Photogrammetric Week '13 Fritsch D, ed233–246 Berlin: Wichmann Herbert.

[b20] Seitz S. M. Curless B. Diebel J. Scharstein D. Szeliski R. (2006). A comparison and evaluation of multi-view stereo reconstruction algorithms. Proceedings of the Conference on Computer Vision and Pattern Recognition 519–528 IEEE Computer Society.

[b21] Sellers W. I. Crompton R. H. (1994). A system for 2- and 3-D kinematic and kinetic analysis of locomotion, and its application to analysis of the energetic efficiency of jumping in prosimians. Z. Morphol. Anthropol. 80, 99–108.

[b22] Sellers W. I. Hill R. A. Logan B. S. (2007). An agent-based model of group decision making in baboons. Philos. Trans. R. Soc. B 362, 1699–1710 10.1098/rstb.2007.2064 PMC2440783 17428770

[b23] Sockol M. D. Raichlen D. A. Pontzer H. (2007). Chimpanzee locomotor energetics and the origin of human bipedalism. Proc. Natl. Acad. Sci. USA 104, 12265–12269 10.1073/pnas.0703267104 17636134 PMC1941460

[b24] Taylor G. K. Bacic M. Bomphrey R. J. Carruthers A. C. Gillies J. Walker S. M. Thomas A. L. (2008). New experimental approaches to the biology of flight control systems. J. Exp. Biol. 211, 258–266 10.1242/jeb.012625 18165253

[b25] Thorpe S. K. S. Holder R. L. Crompton R. H. (2007). Origin of human bipedalism as an adaptation for locomotion on flexible branches. Science 316, 1328–1331 10.1126/science.1140799 17540902

[b26] Tobalske B. Dial K. (1996). Flight kinematics of black-billed magpies and pigeons over a wide range of speeds. J. Exp. Biol. 199, 263–280. 9317775 10.1242/jeb.199.2.263

[b27] Triggs B. McLauchlan P. F. Hartley R. I. Fitzgibbon A. W. (2000). Bundle adjustment – a modern synthesis. Lecture Notes in Computer Science 1883, 298–372 10.1007/3-540-44480-7_21

[b28] Watson J. Payne R. Chamberlain A. Jones R. Sellers W. I. (2009). The kinematics of load carrying in humans and great apes: implications for the evolution of human bipedalism. Folia Primatol. (Basel) 80, 309–328 10.1159/000258646 19923843

[b29] Watson J. C. Payne R. C. Chamberlain A. T. Jones R. Sellers W. I. (2011). The influence of load carrying on gait parameters in humans and apes: implications for the evolution of human bipedalism. Primate Locomotion: Linking Field and Laboratory Research D'Août K Vereecke E E, ed109–134 New York, NY: Springer.

