# Supplementary material for: Markerless 3D motion capture for animal locomotion studies
Source: Biol Open. 2014 Jun 27;3(7):656–68. doi: 10.1242/bio.20148086 (PMC4154302; doi:10.1242/bio.20148086)
Supplement: Supplementary Material [file supp_3_7_656__index.html]

Markerless 3D motion capture for animal locomotion studies — Markerless 3D motion capture for animal locomotion studies — Supplementary Material 

# Markerless 3D motion capture for animal locomotion studies

## bio.20148086 Supplementary Material

**Files in this Data Supplement:**

- Supplementary Material - William Irvin Sellers and Eishi Hirasaki doi: 10.1242/bio.20148086
