# Supplementary material for: Markerless 3D motion capture for animal locomotion studies
Source: Biol Open. 2014 Jun 27;3(7):656–68. doi: 10.1242/bio.20148086 (PMC4154302; doi:10.1242/bio.20148086)
Supplement: Supplementary Material [file supp_bio.20148086_bio.20148086-s1.pdf]

Biology Open (2014) 000, 1–13 doi:10.1242/bio.20148086

Supplementary Material

William Irvin Sellers and Eishi Hirasaki doi: 10.1242/bio.20148086

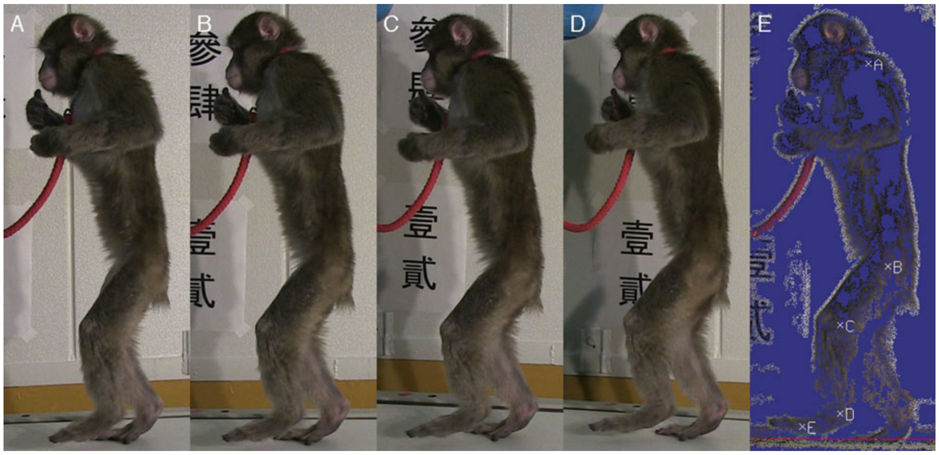

Fig. S1. Japanese macaque walking bipedally on a treadmill. Four camera views (A–D) of a Japanese macaque walking bipedally on a treadmill. These images are cropped (250×600) from the full field of view of the camera (1280×720). (E) The 3D reconstruction generated from these images.

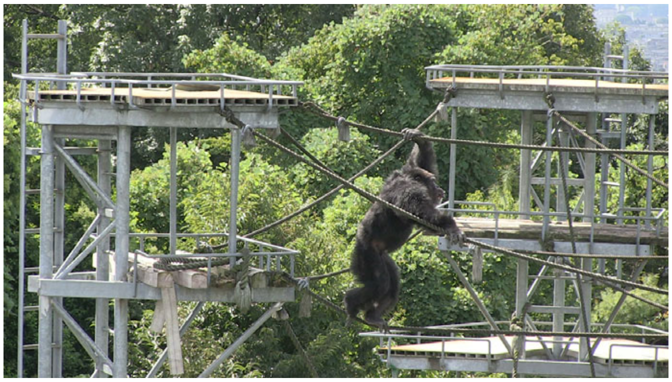

Fig. S2. Full screen still image from one of the cameras used to produce the 3D reconstruction of the bipedally walking chimpanzee (1920×1080).

Biology Open (2014) 000, 1–13 doi:10.1242/bio.20148086

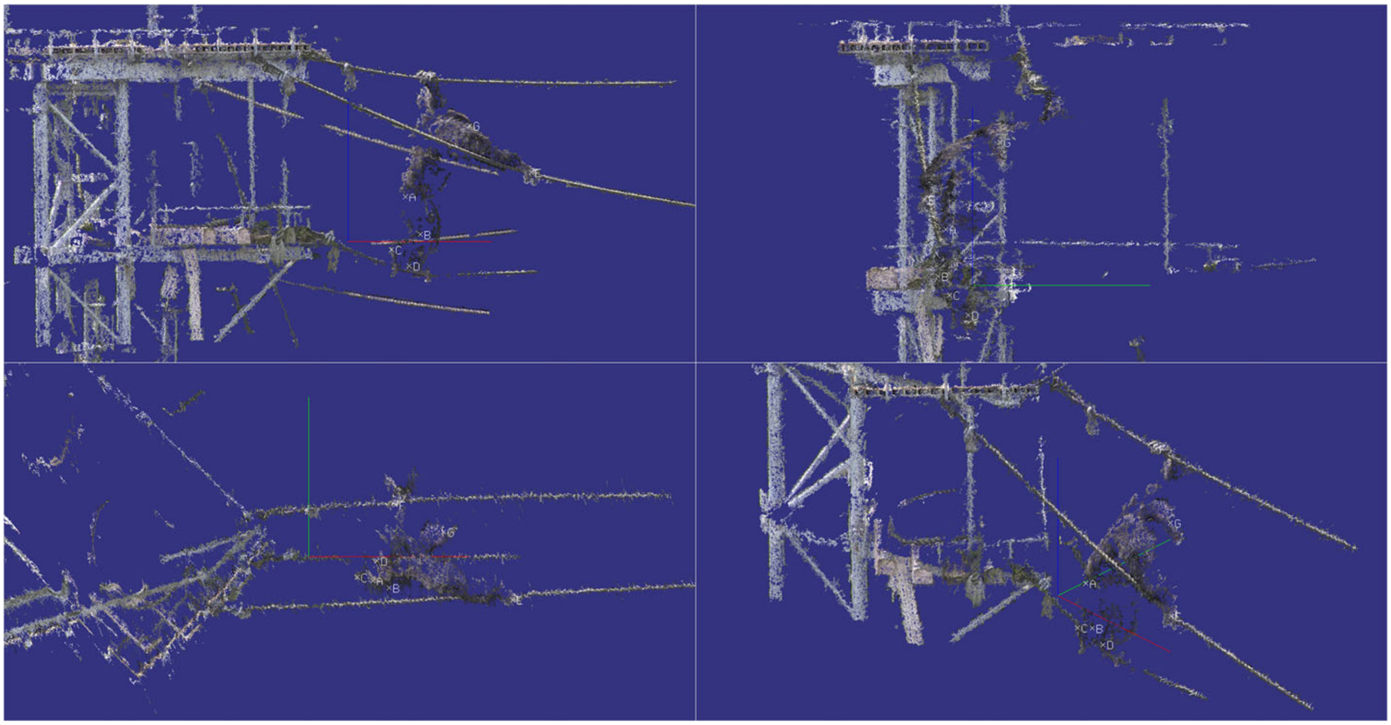

Fig. S3. Screen shot from CloudDigitiser showing the 3D reconstruction of the bipedally walking chimpanzee from the X, Y, Z directions and from an oblique view.

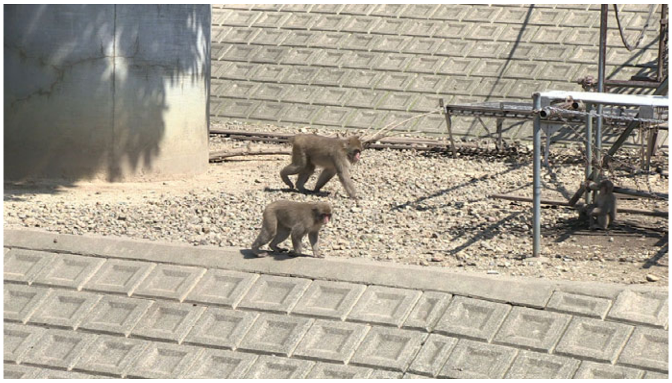

Fig. S4. Full screen still image from one of the cameras used to produce the 3D reconstruction of the Japanese macaque group movements (1920×1080).

Biology Open (2014) 000, 1–13 doi:10.1242/bio.20148086

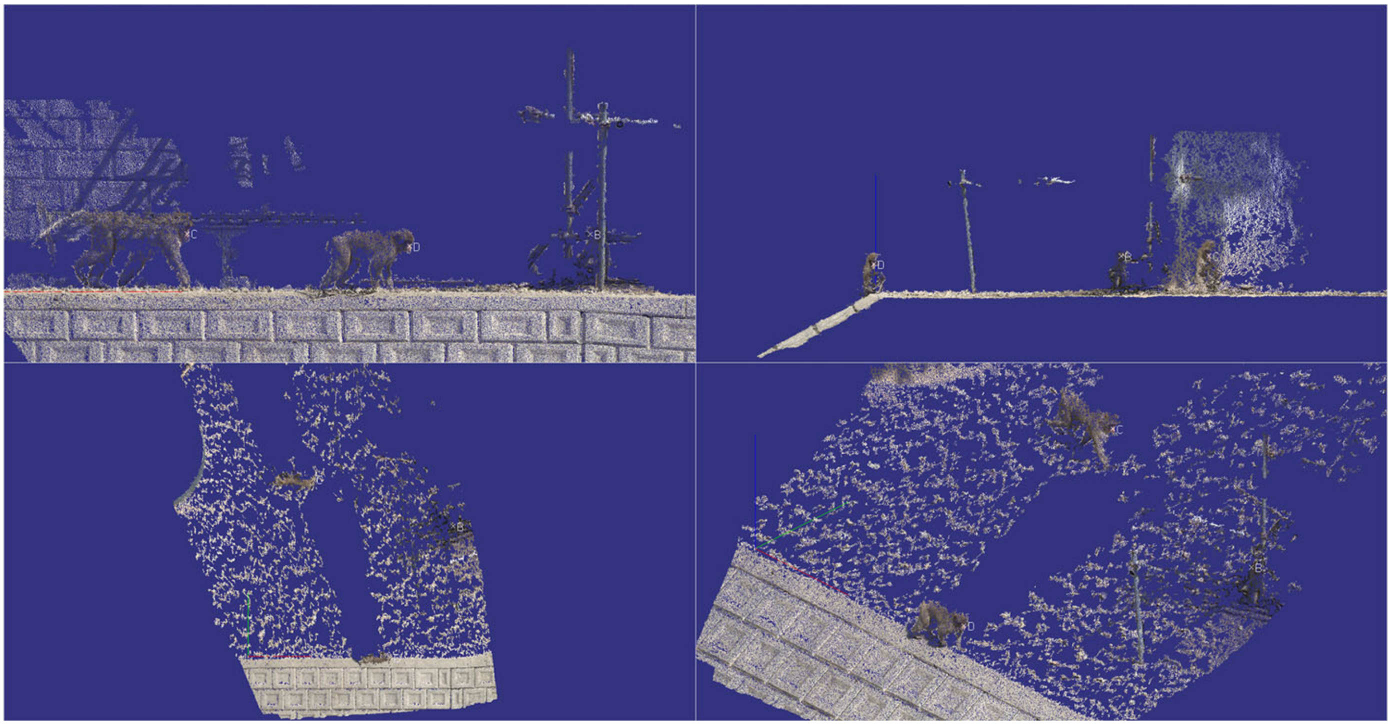

Fig. S5. Screen shot from CloudDigitiser showing the 3D reconstruction of the Japanese macaque group movements from the X, Y, Z directions and from an oblique view.

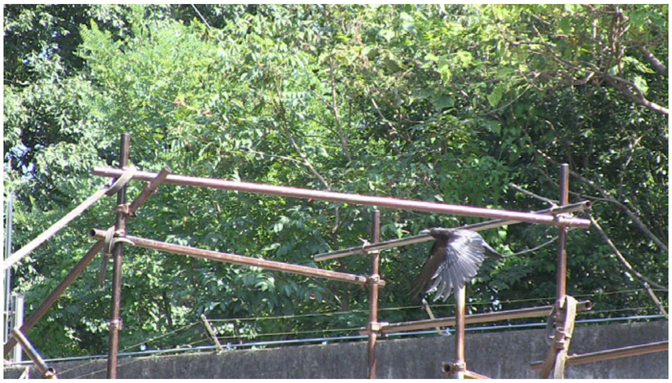

Fig. S6. Full screen still image from one of the cameras used to produce the 3D reconstruction of the crow in flight (1920×1080).

Biology Open (2014) 000, 1–13 doi:10.1242/bio.20148086

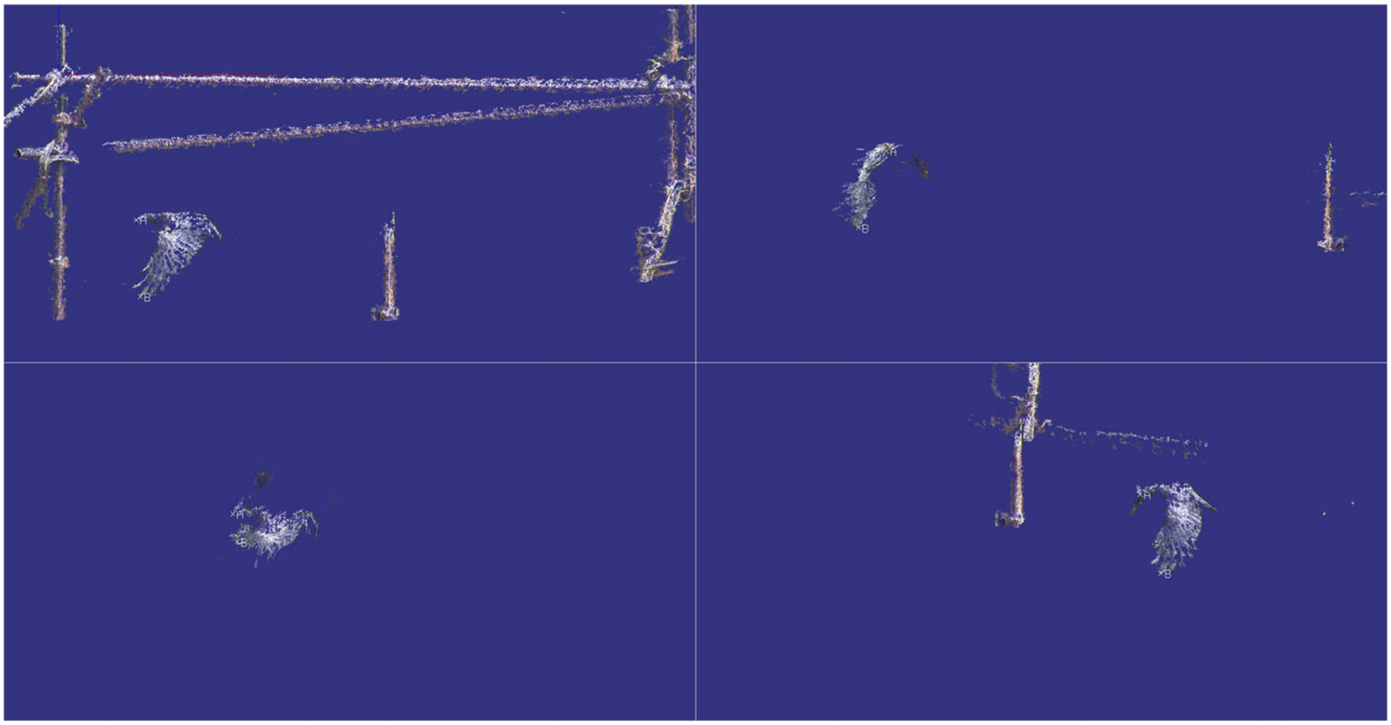

Fig. S7. Screen shot from CloudDigitiser showing the 3D reconstruction of the crow in flight from the X, Y, Z directions and from an oblique view.

Biology Open
